# An Adaptive Unscented Kalman Filter for the Estimation of the Vehicle Velocity Components, Slip Angles, and Slip Ratios in Extreme Driving Manoeuvres

**DOI:** 10.3390/s24020436

**Published:** 2024-01-10

**Authors:** Aymen Alshawi, Stefano De Pinto, Pietro Stano, Sebastiaan van Aalst, Kylian Praet, Emilie Boulay, Davide Ivone, Patrick Gruber, Aldo Sorniotti

**Affiliations:** 1 Centre for Automotive Engineering, University of Surrey, Guildford GU2 7XH, UK; a.alshawi@surrey.ac.uk (A.A.); p.stano@surrey.ac.uk (P.S.); p.gruber@surrey.ac.uk (P.G.); 2 McLaren Automotive, Woking GU21 4YH, UK; stefano.depinto@mclaren.com; 3 Tenneco Automotive, 3800 Sint-Truiden, Belgium; sebastiaan.vanaalst@driv.com (S.v.A.); kpraet@driv.com (K.P.); eboulay@driv.com (E.B.); 4 Independent Researcher, 21100 Varese, Italy; davide.ivone@gmail.com; 5 Departement of Mechanical and Aerospace Engineering, Politecnico di Torino, 10129 Turin, Italy

**Keywords:** unscented Kalman filter, sideslip angle estimation, vehicle speed estimation, slip ratio estimation, state estimation, covariance matrix adaptation

## Abstract

This paper presents a novel unscented Kalman filter (UKF) implementation with adaptive covariance matrices (ACMs), to accurately estimate the longitudinal and lateral components of vehicle velocity, and thus the sideslip angle, tire slip angles, and tire slip ratios, also in extreme driving conditions, including tyre–road friction variations. The adaptation strategies are implemented on both the process noise and measurement noise covariances. The resulting UKF ACM is compared against a well-tuned baseline UKF with fixed covariances. Experimental test results in high tyre–road friction conditions show the good performance of both filters, with only a very marginal benefit of the ACM version. However, the simulated extreme tests in variable and low-friction conditions highlight the superior performance and robustness provided by the adaptation mechanism.

## 1. Introduction

The latest generation of vehicle dynamics controllers, including stability, traction, active steering, and active suspension controllers, as well as automated driving systems, demands increasing amounts of data from the vehicle and its surroundings [1,2,3,4,5]. However, specific vehicle variables, such as the sideslip angle, tyre slip ratios and slip angles, vehicle speed, and tyre–road friction level, cannot be robustly and cost-effectively measured for mass production implementations, and therefore must be estimated. While the data measurements and estimations used since the 1990s for vehicle stability control (VSC) [6] are sufficient for rather simple rule-based algorithms, operating only in emergency scenarios, the progress in high-performance vehicle controllers, implying more frequent and smooth interventions, has increased the demand for more accurate information on the vehicle variables [7,8]. For example, reliable vehicle speed and slip ratio estimations are crucial for effective wheel slip control through actuators capable of continuously and seamlessly modulating the wheel torque levels [9].

In this context, machine learning methods have gained significant attention [10,11,12,13,14,15,16]. These algorithms offer enhanced adaptability to unpredictable situations as they are not based on predefined models, and tend to exhibit increased robustness in scenarios where the accurate modelling of the system dynamics is challenging. Although the resulting agents typically demand less computational power than model-based approaches, their practical implementation requires a very substantial amount of training data [16,17,18]. As mentioned in [18], ‘constructing a complete dataset that can cover all driving conditions is extremely difficult if not impossible’. As a consequence, although machine learning approaches are likely to be more diffusely implemented for state estimation in the near future, Kalman filters are still widely adopted tools to achieve the real-time estimation of vehicle dynamics variables, starting from the measurements from multiple on-board sensors [19].

Several algorithmic solutions have been proposed to account for the vehicle system nonlinearities in the internal model of the filter. This can be typically achieved through extended Kalman filters (EKFs), which imply a linearisation at each time step, or unscented Kalman filters (UKFs), which directly use nonlinear models. Comparative studies of EKF and UKF performance for the same vehicle model, e.g., see [16,20,21,22,23,24], highlight the superiority of UKFs, towards which there has been a progressive shift [17].

In terms of vehicle models embedded in the filters, the literature has assessed a wide range of options. For instance, within a complex estimation architecture for automated vehicles, reference [25] implemented a Kalman filter for sideslip angle estimation, using a 2-degree-of-freedom (2-DoF) linear bicycle model, considering the yaw and sideslip dynamics. This setup performed well during normal driving; however, it lost accuracy at high lateral accelerations or in critical transients. A possible solution to such limitation is the adoption of a double-track vehicle model (DTM), which has become rather widespread [26]. For example, the DTMs within the filters in [21,27,28] include the lateral tyre force nonlinearities, which are modelled through the Pacejka magic formula or the Dugoff formulation. The EKF implementation in [29] and the UKFs in [30,31,32] augmented the DTM by including the longitudinal vehicle dynamics and wheel dynamics, which resulted in 7-DoF vehicle models, enabling tyre slip ratio estimation.

In general, there are limited studies assessing Kalman filter performance on low-friction surfaces. References [29,32] are rare exceptions, but their setups are constrained by fixed settings of the filter parametrisation. Additionally, the tests in [29,32] omitted high wheel slip cases, such as wheel spinning and locking, analysing only the sideslip angle estimation.

In most vehicle dynamics filters, vehicle and tyre parameters are assumed to remain constant over time. However, some implementations deal with parametric uncertainties by embedding parallel estimators for the main vehicle parameters, or by concurrently estimating vehicle states and parameters. For example, the Kalman filter in Wenzel et al. [33] was supported by a second Kalman filter, which enabled the update of the vehicle mass, yaw mass moment of inertia, and longitudinal position of the centre of gravity. The algorithms in [30,31,32,34,35] varied the peak tyre–road friction coefficient and/or the cornering stiffness during their operation.

Similarly to the vehicle and tyre parameters, the EKF/UKF covariance matrices usually remain constant over time, and can be tuned through Bayesian optimisations [36] to find desirable trade-offs for a catalogue of manoeuvres. However, the effectiveness of the resulting filter is limited to specific conditions, and significant issues can occur during real-world operation. An effective approach to improve predictability and handle situations where the uncertainty characteristics of the system change over time is to employ adaptive covariance matrices [37,38,39,40,41,42,43,44]. The adaptation approaches can either be based on theoretical algorithms, e.g., using fading factors to give higher weight to the last filter iterations, or on covariance definition rules, e.g., imposed through fuzzy logic algorithms or other heuristics benefitting from the understanding of vehicle dynamics.

References [37,38,39,40] present adaptation strategies for the measurement noise covariances. Specifically, an adaptation scheme limited to the wheel speed measurement noise covariances was used in the EKF in [37], to target high wheel slip conditions, based on empirical rules. In such conditions, although the wheel speed and longitudinal acceleration measurements processed by the typical vehicle stability control unit are accurate, reliable, and without outliers, it is usually better to vary the weight associated with the angular wheel speed and longitudinal acceleration measurements, to prioritise the latter for vehicle speed estimation. Among the theory-based algorithms, references [38,39] used the moving window estimation method, while reference [40] relied on a maximum a posteriori algorithm. However, the assessment was limited to high-friction conditions, and no adaptation was applied to the process noise covariances.

By employing a comparable method to [40], reference [41] adapted the process and measurement noise covariances. Although this algorithm can offer stability in scenarios with noisy or incomplete data, its complexity could become critical when applied to filters characterised by vehicle models with higher number of degrees of freedom (DoFs). Reference [42] discusses a normalized innovation squared (NIS) algorithm to adaptively change both process and measurement noise covariances, by comparing the difference between predicted and observed measurements to the predicted covariance of measurement noise. However, alongside its computational complexity, the NIS approach requires precise tuning, and lacks robustness when encountering model inaccuracies. Reference [43] proposes an exponential decay adaptive unscented filter, in which the exponential decay factor helps assign different weights or decay rates to older and newer measurements, allowing the filter to adaptively weigh recent information more heavily than older data. Nevertheless, the algorithm lacks flexibility in accommodating rapid variations in state variables, resulting in compromised estimation accuracy, as shown for a sinusoidal steering manoeuvre. The authors conclude that “…however, the average weighting method and the general exponential weighting method are not flexible enough. As a result, when the state variable changes rapidly, the noise cannot be accurately estimated, and the state variable cannot be evaluated more accurately…. The proposed method has some improvement effect compared with the original method, but the effect is not significant enough in some conditions, which is a defect of the proposed method”. In summary, the adaptation solutions in [41,42,43] primarily relied on the measurement noise level, and were not particularly effective for swift variations of states or tyre–road friction parameters, or for incorporating physics-based heuristics. Additionally, the proposed filters used simplified models, neglecting the wheel dynamics, which are of the essence for tyre slip dynamics estimation in extreme conditions. Finally, the evaluation was limited to simulations with constant or slowly varying torque demands, without significant wheel slip events.

Interestingly, van Aalst et al. [44] combined parameter and covariance adaptations. In fact, the cornering stiffness values are adapted with a random walk model, while the filter covariances are adjusted according to the operating conditions of the vehicle, by using vehicle-dynamics-derived heuristics. The resulting scheme can thus effectively account for uncertainties and nonlinearities across a wide range of excitation inputs. However, the algorithm, based on an EKF approach, is not designed for longitudinal speed and slip ratio estimation, and is assessed only in high-friction conditions.

A recent trend is the use of combinations of state estimators, e.g., through so-called fusion architectures that embed both kinematic and dynamic vehicle models. In fact, a kinematic model is not affected by uncertain vehicle parameters or tyre behaviour, and can be particularly effective in transient conditions and for large excitation magnitudes. On the contrary, during normal vehicle operation, dynamic model-based filters provide better performance. For example, the solution in [45] combined a consensus Kalman filter, using a dynamic vehicle model, and a kinematic model-based Kalman filter. The architecture was applied to an automated vehicle, with the two filters receiving heading and velocity errors determined through a global navigation satellite system (GNSS) and a reduced inertial navigation system (R-INS). A weighting scheme based on the lateral excitation level fused the two outputs, bringing improved estimation accuracy during critical driving conditions. In the architecture in [46], an algorithm adaptively determined the cornering stiffness of a single-track model for sideslip angle estimation during quasi-steady-state cornering, through a proportional integral (PI) controller. Additionally, a kinematic approach in the form of an EKF was employed for transients and large excitation magnitudes. Similarly to [45], a weighting strategy combined the filter outputs. Whilst the resulting fusion architectures were assessed through a wide range of experimental data, neither paper embedded tests on low-μ surfaces, or incorporated the wheel dynamics, i.e., the tyre slip ratios could not be accurately estimated during wheel slip control transients. Furthermore, these approaches are more complex to tune than a single estimator, and thus, instead of fusing two estimators to maximise performance, a covariance adaptation strategy on a single estimator may be more practical.

In summary, the available literature lacks:

- Adaptive dynamic model-based Kalman filters that concurrently vary the process and measurement noise covariances through vehicle-dynamics-derived heuristics. These should be defined as a function of relevant error variables, based on the difference between the measured and estimated outputs, with the specific scope of enhancing performance in highly dynamic conditions, including significant longitudinal tyre slip variations induced by wheel torque or tyre–road friction transients. These scenarios, unlike the current adaptive implementations, require internal models accounting for the wheel dynamics.
- The assessment of the sideslip angle, velocity and tyre slip estimation performance benefit of adaptive Kalman filters in both high- and low-friction conditions, including μ-jump tests, and manoeuvres with very high levels of longitudinal and lateral slip.

This paper targets the identified gap, with the following contributions:

- A UKF implementation with vehicle-dynamics-based adaptive formulations for the following: (a) the wheel speed measurement covariances, based on variables that are robustly representative of the longitudinal tyre slip condition; (b) the tyre–road friction coefficient process noise covariance, based on error variables depending on the longitudinal and lateral accelerations; and (c) the process noise covariances of the yaw rate and the longitudinal and lateral velocity components, based on the estimation errors with respect to the available relevant measurements.
- The experimental validation of the UKF with adaptive covariance matrices, referred to as UKF ACM, along extreme high-friction manoeuvres, including significant longitudinal and lateral accelerations.
- The validation of UKF ACM through a high-fidelity and experimentally validated model, in conditions with very low tyre–road friction, including μ-jumps.
- The comparison of UKF ACM with a baseline UKF with fixed covariance values that are well calibrated.

The remainder is organised as follows: Section 2 introduces the case study vehicle and simulation models; Section 3 presents the proposed UKF implementations; Section 4 deals with the case study manoeuvres and relevant key performance indicators; Section 5 reports and critically analyses the experimental and simulation results; finally, Section 6 draws the main conclusions.

## 2. Case Study Vehicle and Associated Models

### 2.1. Reference Vehicle

**Figure 1:**
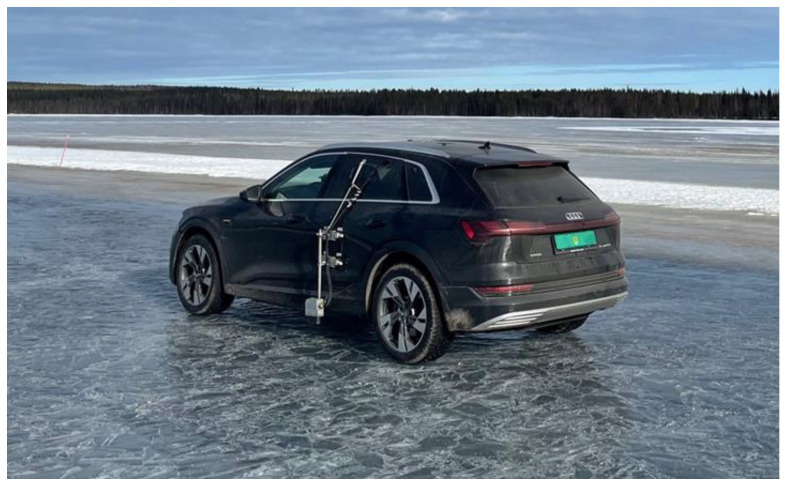
The case study Audi e-tron at the Ebbenjarka testing facility (Sweden), including the Kistler Correvit S-350 sensor, providing accurate measurements of the velocity components.

The case study was an Audi e-tron sport utility vehicle prototype, used as one of the demonstrators of the European Horizon 2020 EVC1000 project. The vehicle has two on-board electric motors, one per axle, connected to the wheels via a single-speed transmission and mechanical differential. The main vehicle parameters are in Table 1. Figure 1 shows a photograph of the vehicle, with a Kistler Correvit S-350 sensor mounted on the rear left door. The sensor provides accurate measurements of the longitudinal and lateral components of vehicle velocity, and thus sideslip angle, which are recorded for validating the values estimated by the filters. The vehicle also includes the typical factory-installed measuring equipment, e.g., an inertial measurement unit (IMU), as well as steering wheel angle, individual wheel speed, accelerator pedal position, and tandem master cylinder pressure sensors. The considered filters only used the conventional production vehicle measurements as inputs.

### 2.2. High-Fidelity Vehicle Simulation Model

A high-fidelity vehicle simulation model was implemented with the CarMaker tool by IPG, for the simulation-based assessment of the UKF performance. The model includes the six DoFs of the sprung mass, the four DoFs associated with the unsprung mass motions, and the four wheel rotations, and considers the associated dynamic couplings. The suspension elasto-kinematic characteristics and nonlinearities associated with the shock absorbers and bump stops were also accounted for. Tyre behaviour was emulated with the Pacejka magic formula [47] including relaxation effects. The model parameters were directly provided by the involved car manufacturer, which was part of the EVC1000 initiative.

### 2.3. Internal Model of the Filters

**Figure 2:**
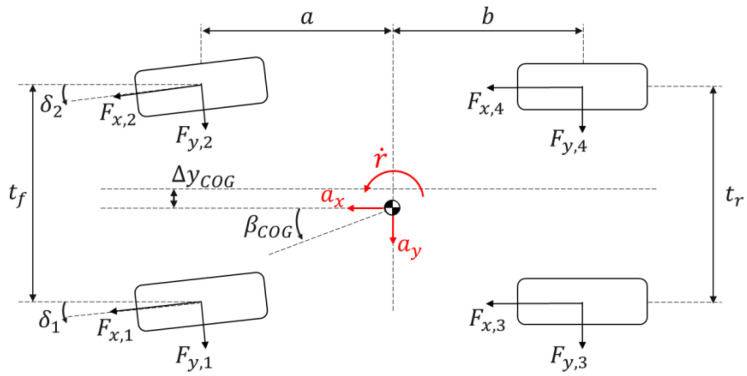
Top view schematic of the internal model of the estimator with indication of the main variables and parameters.

The internal model embedded in the UKFs was specifically designed to achieve its intended objectives, i.e., it includes only the essential DoFs for estimating the sideslip angle, vehicle speed, slip angles, and slip ratios, in order to achieve computational efficiency and real-time implementability. Therefore, the selected formulation is a nonlinear 7-DoF model, considering the longitudinal, lateral, and yaw dynamics of the vehicle, in addition to the rotational dynamics of each of the four wheels. The schematic in Figure 2 highlights the key parameters and variables, together with their sign conventions, according to the ISO standard [48]. The internal model was developed by Mazzilli et al. [49]; hence, its detailed derivation will not be included here.

The primary governing equations of motion are the following:

- Longitudinal force balance
  (1) $$a_{x}={\dot{v}}_{x}-v_{y}r=\frac{1}{m_{tot}}\left[\sum_{i=1}^{2}\left(F_{x,i}cos\delta_{i}-F_{y,i}sin\delta_{i}\right)+\sum_{i=3}^{4}\left(F_{x,i}\right)-F_{drag}\right]$$
- Lateral force balance
  (2) $$a_{y}={\dot{v}}_{y}+v_{x}r=\frac{1}{m_{tot}}\left[\sum_{i=1}^{2}\left(F_{x,i}sin\delta_{i}+F_{y,i}cos\delta_{i}\right)+\sum_{i=3}^{4}\left(F_{y,i}\right)\right]$$
- Yaw moment balance
  (3) $$\begin{array}{c} \dot{r}=\frac{1}{I_{z}}[a\overset{2}{\underset{i=1}{\sum }}\left(F_{x,i}sin\delta_{i}+F_{y,i}cos\delta_{i}\right)+(\frac{t_{f}}{2}-∆y_{COG})(F_{y,1}sin\delta_{1}-F_{x,1}cos\delta_{1})\\ \;-(\frac{t_{f}}{2}+∆y_{COG})(F_{y,2}sin\delta_{2}-F_{x,2}cos\delta_{2})-(\frac{t_{r}}{2}-∆y_{COG})F_{x,3}\\ \;+(\frac{t_{r}}{2}+∆y_{COG})F_{x,4}-b\overset{4}{\underset{i=3}{\sum }}F_{y,i}+\overset{4}{\underset{i=1}{\sum }}M_{z,i}] \end{array}$$
- Wheel moment balance
  (4) $$I_{weq,i}{\dot{\omega }}_{i}=M_{EM,i}-M_{B,i}-F_{x.i}R_{l,i}-M_{y,i}$$
  where the increasing values of the integer i, with i= 1,…, 4, used as a subscript, refer to the front left, front right, rear left, and rear right vehicle corners; $a_{x}$ and $a_{y}$ are the longitudinal and lateral accelerations; $v_{x}$ and $v_{y}$ are the longitudinal and lateral components of the vehicle velocity; $F_{x,i}$ and $F_{y,i}$ are the longitudinal and lateral tyre forces; $\delta_{i}$ is the steering angle of the i-th wheel; r is the yaw rate; $F_{drag}$ is the aerodynamic drag force; $∆y_{COG}$ indicates the lateral position of the centre of gravity with respect to the plane of symmetry of the vehicle; $M_{z,i}$ is the tyre self-aligning moment; $I_{weq,i}$ is the equivalent mass moment of inertia of the wheel; ${\dot{\omega }}_{i}$ is the angular wheel acceleration; $M_{EM,i}$ is the electric motor torque level referred to the i-th corner, computed from the measured motor current, the gearbox and final drive ratios, as well as the respective efficiencies; $M_{B,i}$ indicates the braking torque at the individual corner, which is estimated from the measured tandem master cylinder pressure; $R_{l,i}$ is the laden tyre radius; and $M_{y,i}$ is the rolling resistance moment.

The model computes the longitudinal and lateral load transfers through steady-state equations based on $a_{x}$ and $a_{y}$. The vehicle sideslip angle at the centre of gravity, $\beta_{COG}$, which is one of the key variables to be estimated, is given by:

(5) $$\beta_{COG}=\tan^{-1}\left(\frac{v_{y}}{v_{x}}\right)$$

Similarly, the classical kinematic equations are used for the tyre slip ratios, $\sigma_{x,i}$, and slip angles, $\alpha_{i}$, which are modified according to the analyses in [50,51], to ensure stability and realism also during low-speed operation. The longitudinal and lateral tyre forces are obtained through a simplified version of the magic formula (version 5.2, see [47]).

In summary, the formulation of the internal model of the filter is totally independent from the CarMaker model in Section 2.2. The very substantial differences in key aspects of the models (e.g., number of DoFs, load transfer formulations, magic formula version) also imply different model parametrisations. To make the UKF performance assessment more realistic and conservative, the model calibrations, based on the available vehicle data, did not involve any further optimisation to match the collected experimental results. The diversity in model arrangements and tuning setups makes the following simulation analyses realistic and indicative of the operational robustness of the UKFs.

### 2.4. Experimental Validation of the Models

The CarMaker and internal models have been experimentally validated through series of quasi-steady-state and transient tests in high-friction conditions, carried out during the EVC1000 project. Further experimental data was gathered on the very low-friction ice tracks at the Ebbenjarka testing facility in Sweden, which was characterised by variability and unpredictability of the surface conditions.

**Figure 3:**
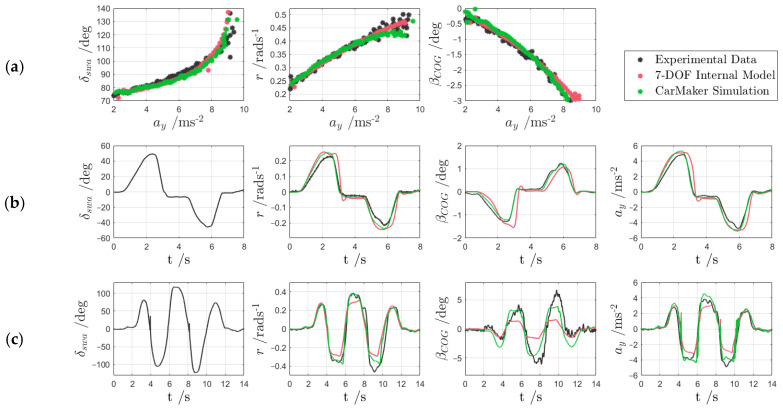
Examples of experimental validation of the CarMaker model and the internal UKF model on (**a**) a 40 m radius skid pad on dry tarmac; (**b**) a sinusoidal steering manoeuvre on dry tarmac, from ~80 kmh^−1^; and (**c**) a sinusoidal steering test at ~40 kmh^−1^ on an icy surface (t is time).

Figure 3 reports a sample of the validation results, referring to (a) a quasi-steady-state 40 m radius skid pad manoeuvre on dry tarmac; (b) a sinusoidal steering manoeuvre from an initial speed of ~80 kmh^−1^ and constant accelerator pedal position; and (c) a sinusoidal steering manoeuvre at ~40 kmh^−1^ on an icy surface.

The three sets of data—the experimental results and the simulation results from the two models—highlight that the CarMaker model provided a consistently good match for experiments (a–c), and therefore could be considered a reliable tool for UKF performance assessment across a wide range of conditions, including very low tyre–road friction. On the contrary, although performing well in the high-μ tests, the UKF models severely underestimated the sideslip angle magnitude in the low-friction test (c), which highlights the necessity of the UKF ACM.

## 3. Adaptive Unscented Kalman Filter Architecture

### 3.1. Filter Architecture and Strategy

**Figure 4:**
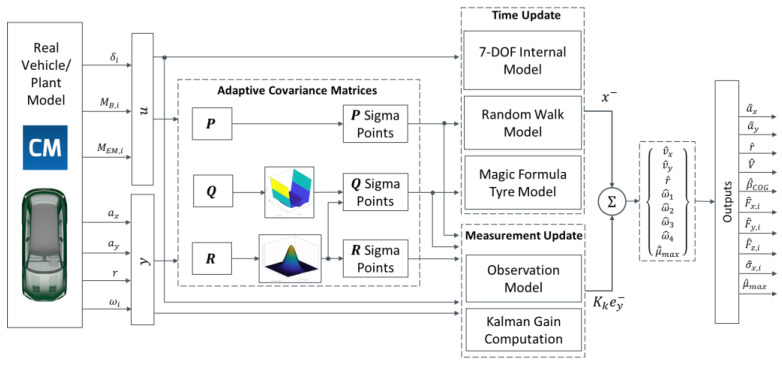
Overview schematic of the presented UKF ACM state estimator.

Figure 4 is an overview schematic of the filter architecture, which was implemented and assessed in real-time on a dSPACE MicroAutoBox II unit (900 MHz, 16 Mb flash memory), with a 10 ms sampling time.

The system state, input, and output (corresponding to the available measurements) vectors, respectively, x, u, and y, are:

(6) $$x=\left\{\;v_{x}\;v_{y}\;r\;\omega_{i}\;{\bar{\mu }}_{max}\right\}\;u=\left\{\;\delta_{i}\;M_{EM,i}\;M_{B,i}\;\right\}\;y=\left\{\;a_{x}\;a_{y}\;r\;\omega_{i}\;\right\}$$

As per [49], the average peak tyre–road friction coefficient (${\bar{\mu }}_{max}$) of the four tyres is one of the states, and is estimated via a random walk model in the time update step. ${\bar{\mu }}_{max}$ is used as an additional degree of freedom to provide more accurate output estimations through the UKF, such as those of the vehicle speed and sideslip angle, as opposed to attaining an accurate friction coefficient estimation.

The presented estimators are based on the UKF algorithm [24], which operates according to two main steps:

1. The time update (or prediction) step, in which a predicted state vector $x_{k}^{-}$, also known as the a priori state vector, where k is the time step, is computed by using the previous state estimate and the internal nonlinear vehicle model. $x_{k}^{-}$ is calculated by estimating the mean of so-called sigma points, which approximate the mean and covariance of the system, representing the state estimate and its associated uncertainty. In this step, the process noise covariance matrix, Q, is used to influence the propagation of the generated sigma points. High values of the elements of Q indicate significant uncertainty and low confidence in the internal model dynamics, which consequently affects the a priori prediction [52].
2. The measurement update (or correction) step, in which $x_{k}^{-}$ is corrected to produce the a posteriori state vector estimate, $x_{k}^{+}$, by multiplying the error between the predicted measurements and the real measured data, also known as the innovation, $e_{y,k}^{-}$, by the Kalman gain, $K_{k}$, the calculation of which is beyond the scope of this paper:
  (7) $$x_{k}^{+}=x_{k}^{-}+K_{k}e_{y,k}^{-}$$

The measurement noise covariance matrix, R, which represents the uncertainty in the vehicle sensors, is used to influence the spread of a second set of sigma points, generated similarly to those in step 1., and is also directly involved in the calculation of $K_{k}$. Within the UKF algorithms, larger values of the elements of R imply reduced significance associated with the measurements.

A third covariance matrix, namely the state covariance matrix P, which quantifies the uncertainty in the state vector $x_{k}$, is used in both steps of the UKF algorithm. While P is a full matrix that is updated iteratively at each time step according to the UKF equations, Q and R are diagonal matrices to reduce the computational load, under the assumption that the process and measurement noise sources are independent from each other. Hence, each element of Q and R correlates to the uncertainty of a state or measurement. In the specific implementation, Q and R are given by:

(8) Q=diag[QvxQvyQrQω1Qω2Qω3Qω4Qμ¯max]

(9) R=diag[RaxRayRrRω1Rω2Rω3Rω4]

where the subscripts in the notations of the elements of Q indicate the related state, while the subscripts of the components of R refer to the relevant measurement. Q and R are typically kept constant. In fact, well-calibrated fixed-covariance UKFs are sufficient in the majority of real-world cases, but there are exceptional conditions where this is not true, and thus an ACM setup is beneficial to robustness and convergence. In this study, two UKF variants are presented: (i) the baseline UKF, simply referred to as UKF, with fixed values of the matrices; and (ii) UKF ACM, including the adaptation of the elements of Q and R indicated in the boxes in (8) and (9).

The process and measurement noise covariances of the baseline UKF were obtained via an optimisation routine, minimising a cost function based on the error between the UKF estimated outputs and the measurements, see [49], which was computed from test results obtained along the high-friction handling circuit of the Lommel proving ground (Belgium).

### 3.2. Wheel Speed Measurement Noise Covariance Adaptation

In UKFs with constant covariances, the accuracy of the $v_{x}$ estimation typically worsens as the magnitude of the longitudinal tyre slip ($\sigma_{x}$) increases. In fact, when the tyres tend to lock or spin, the wheel speed measurements, despite remaining accurate and robust, are not effective for straightforward vehicle speed estimation. In these conditions, it is more effective to prioritise (i) the speed estimate from the nonlinear internal vehicle model of the time update step, which embeds the tyre slip dynamics of each corner, if the tyre–road friction factor is correctly estimated; and/or (ii) the $a_{x}$ measurement, which, through integration in the time domain, could ideally directly bring the speed profile.

To achieve the desired effect, UKF ACM includes an adaptation of the R matrix values for the four wheel speed measurements, i.e., $R_{\omega_{i}}$, at each time step, according to:

(10) $$R_{\omega_{i}}=R_{\omega ,min}+\left(R_{\omega ,max}-R_{\omega ,min}\right){\left(1-\epsilon_{\omega_{i}}\right)}^{2}$$

where $R_{\omega ,min}$ and $R_{\omega ,max}$ are the minimum and maximum values for $R_{\omega_{i}}$, and $\epsilon_{\omega_{i}}$ is an adaptive weight.

The weight $\epsilon_{\omega_{i}}$ varies according to the following bivariate normal distribution:

(11) $$\epsilon_{\omega_{i}}=e^{-\frac{1}{2}\;\left[{\left(\frac{\bar{a_{x,\omega_{i}}-a_{x}}}{\sigma_{a}}\right)}^{2}+{\left(\frac{\bar{v_{x,\omega_{i}-{\hat{v}}_{x,\omega }}}}{\sigma_{v}}\right)}^{2}\right]}$$

where $a_{x}$ is the measured longitudinal acceleration; ${\hat{v}}_{x,\omega }$ is a dead reckoning estimated longitudinal speed based on the average of the four wheel speeds; $\sigma_{a}$ and $\sigma_{v}$ are tuneable standard deviation parameters for the longitudinal acceleration and speed; and $a_{x,\omega_{i}}$ and $v_{x,\omega_{i}}$ are the longitudinal acceleration and velocity of the respective corner, obtained from the measured i-th wheel speed and transformed to the vehicle centre of gravity, under the simplifying assumption of zero longitudinal tyre slip:

(12) $$v_{x,\omega_{1}}=-r\frac{t_{f}}{2}+\omega_{1}R_{1}\cos\delta_{1}\;v_{x,\omega_{2}}=r\frac{t_{f}}{2}+\omega_{2}R_{2}\cos\delta_{2}\;v_{x,\omega_{3}}=-r\frac{t_{r}}{2}+\omega_{3}R_{3}\;v_{x,\omega_{4}}=r\frac{t_{r}}{2}+\omega_{4}R_{4}\;a_{x,\omega_{i}}={\dot{v}}_{x,\omega_{i}}$$

where $R_{i}$ is the rolling radius of the respective tyre. The bar notation ‘$\bar{⬚}$’ in the numerator of (11) denotes a moving average computed on four samples with 10 ms discretisation. The term $\bar{a_{x,\omega_{i}}-a_{x}}$ is an indicator of the absolute rate of variation of the tyre slip in the considered corner, since the reference is represented by the longitudinal vehicle acceleration measurement, while the term $\bar{v_{x,\omega_{i}}-{\hat{v}}_{x,\omega }}$ highlights the wheels with significantly different longitudinal slip from the average value among the corners. Hence, from the adaptation mechanism perspective, the absence of criticality corresponds to conditions of rather uniform longitudinal tyre slip distribution among the corners, and the absence of significant longitudinal slip rates. During the filter implementation phase, it was extensively verified through simulations and experiments that the combination of the two terms is effective and robust in detecting the critical corners from the viewpoint of the slip ratio dynamics.

**Figure 5:**
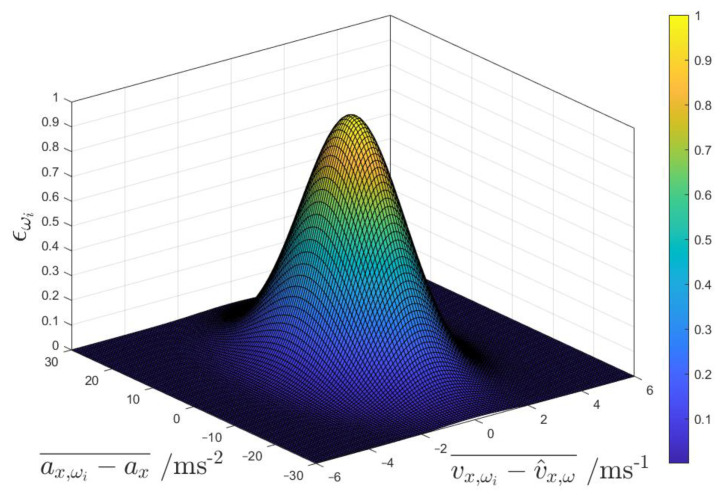
Plot of $\epsilon_{\omega_{i}}$ as a function of $\bar{a_{x,\omega_{i}}-a_{x}}$ and $\bar{v_{x,\omega_{i}}-{\hat{v}}_{x,\omega }}$.

Figure 5 shows the adopted $\epsilon_{\omega_{i}}$ distribution as a function of $\bar{a_{x,\omega_{i}}-a_{x}}$ and $\bar{v_{x,\omega_{i}}-{\hat{v}}_{x,\omega }}$. $\epsilon_{\omega_{i}}$ varies between 0, indicating the presence of critical slip dynamics, and 1, indicating the absence of detected longitudinal slip criticalities. Based on (10), $\epsilon_{\omega_{i}}=$ 1 corresponds to $R_{\omega ,min}$, i.e., the condition of maximum weight given to the wheel speed measurement, while $\epsilon_{\omega_{i}}=$ 0 implies $R_{\omega ,max}$, i.e., the minimum significance given to the wheel speed signal. The use of a standard deviation approach in (11) enables the capture of the range of potential values and the likelihood of their occurrence within a normal distribution framework. This is particularly useful in the presented adaptation algorithm, in which the two variables are interrelated and exhibit a certain level of correlation in their behaviour [53].

During the filter calibration phase, the parametrisation of the standard deviation coefficients $\sigma_{a}$ and $\sigma_{v}$ was carried out through brute-force testing in manoeuvres implying different levels of excitation of the longitudinal vehicle dynamics. $R_{\omega ,min}$ was set to be equal to the $R_{\omega }$ value that was optimised for the baseline UKF, providing desirable performance for a very wide range of operating vehicle conditions. Instead, $R_{\omega ,max}$ was determined via a dedicated optimization process. By using the value from van Aalst’s research [37] as the initial condition, a multi-objective genetic algorithm was executed with constant $R_{\omega_{i}}$ values, equal among the corners, i.e., $R_{\omega_{1}}=\cdots =R_{\omega_{4}}=R_{\omega }$. The routine minimises an objective function, $J_{b}$, which includes six terms, accounting for the relevant normalized root mean square error (NRMSE) values between the measurements and the filter estimation outputs:

(13) $${arg\;{min}_{R_{\omega }}\;J}_{b}=\sqrt{\frac{1}{N_{s}}\sum_{b}\left(\sum_{p=1}^{N_{s}}{\left[\frac{e_{w,b,p}}{e_{norm,b}}\right]}^{2}\right)}\;\;\;\mathrm{with}\;b=a_{x},v_{x},\omega_{1},\omega_{2},\omega_{3},\omega_{4}$$

where the index b—used both as a main variable and as a subscript—refers to the longitudinal acceleration $a_{x}$, longitudinal speed $v_{x}$, and wheel speeds $\omega_{i}$; $N_{s}$ is the number of samples within the considered manoeuvre; $e_{norm,b}$ is the normalisation factor for the corresponding error variable; and $e_{w,b,p}$ is the weighted estimation error of the sample p, which is given by:

(14) $$e_{w,b,p}=\left\{\begin{array}{cc} W_{1,b}({\hat{b}}_{p}-b_{p}) & if\left|{\hat{b}}_{p}-b_{p}\right|<e_{th,1,b}\\ W_{2,b}({\hat{b}}_{p}-b_{p}) & if\;e_{th,1,b}\leq \left|{\hat{b}}_{p}-b_{p}\right|<e_{th,2,b}\\ W_{3,b}({\hat{b}}_{p}-b_{p}) & if\left|{\hat{b}}_{p}-b_{p}\right|\geq e_{th,2,b} \end{array}\right.$$

where $e_{th,1,b}$ and $e_{th,2,b}$, with $e_{th,1,b}>$ $e_{th,2,b}$*,* are predefined $\left|{\hat{b}}_{p}-b_{p}\right|$ thresholds; and the scaling factors $W_{1,b}$, $W_{2,b}$, and $W_{3,b}$, with ${W_{3,b}\gg W}_{2,b}\gg W_{1,b}$, are set to penalise excessive error values. The optimisation round only covered manoeuvres with extremely critical steady-state and transient longitudinal tyre slip conditions, and the resulting optimal $R_{\omega }$ value was set as $R_{\omega ,max}$. Further trial-and-error calibration of $R_{\omega ,max}$ was implemented at the end of the overall filter adaptation mechanism tuning, by considering the adaptations of the other UKF covariances.

### 3.3. Process Noise Covariance Adaptation

The process noise covariances $Q_{r}$, $Q_{v_{x}}$, and $Q_{v_{y}}$ are characterised by a similar adaptation structure to the wheel speed measurement noise covariances. During the development phase, it was verified that these adaptations tend to improve estimation robustness, especially in extremely critical conditions. For example, this applies to specific counter-steering and drifting manoeuvres, in which the yaw rate and front steering angles often have opposite signs. Hence, the internal model tends to be less accurate, and the UKF must increase its reliance on measured values.

The covariances vary between minimum and maximum set values as functions of $\epsilon_{j}$:

(15) $$Q_{j}=Q_{j,min}+\left(Q_{j,max}-Q_{j,min}\right){\left(1-\epsilon_{j}\right)}^{2}$$

where the subscript j, with j=r, $v_{x}$, $v_{y}$, indicates the relevant state; the minimum and maximum covariance values, $Q_{j,min}$ and $Q_{j,max}$, are computed with a similar procedure to the one used for $R_{min}$ and $R_{max}$; and $\epsilon_{j}$ is calculated based on a Gaussian distribution law:

(16) $$\epsilon_{j}=e^{-\frac{1}{2}{\left(\frac{\bar{\hat{l}-l}}{\sigma_{l}}\right)}^{2}}$$

where the notation l, with l=r, $a_{x}$, $a_{y}$, indicates the relevant measured variable; $\hat{l}$ is the value of the same variable estimated by the UKF; and $\sigma_{l}$ is a tuneable standard deviation parameter. Whilst r is used for the computation of $\epsilon_{r}$, the accelerations $a_{x}$ and $a_{y}$ are adopted for $\epsilon_{v_{x}}$ and $\epsilon_{v_{y}}$, instead of $v_{x}$ and $v_{y}$, as it is preferable to build the adaptation upon the estimation error with respect to measured values such as $a_{x}$ and $a_{y}$.

Extensive testing revealed a significant influence of the measurement covariance for ${\bar{\mu }}_{max}$, i.e., $Q_{{\bar{\mu }}_{max}}$, on the adaptability and rate of change of ${\bar{\mu }}_{max}$, especially in scenarios characterised by high levels of lateral and/or longitudinal tyre slip. Consequently, for this covariance, a different adaptation strategy was developed, using a two-dimensional look-up table, based on two variables related to the lateral and longitudinal dynamics: (a) the moving average of the error between the lateral acceleration value estimated by the UKF and the one measured by the IMU, i.e., $e_{a_{y}}=\bar{{\hat{a}}_{y}-a_{y}}$; and (b) the time derivative of the moving average of the longitudinal acceleration error, ${\dot{e}}_{a_{x}}=d\left(\bar{{\hat{a}}_{x}-a_{x}}\right)/dt$, which is a good indicator of friction-related criticalities of the longitudinal vehicle dynamics.

**Figure 6:**
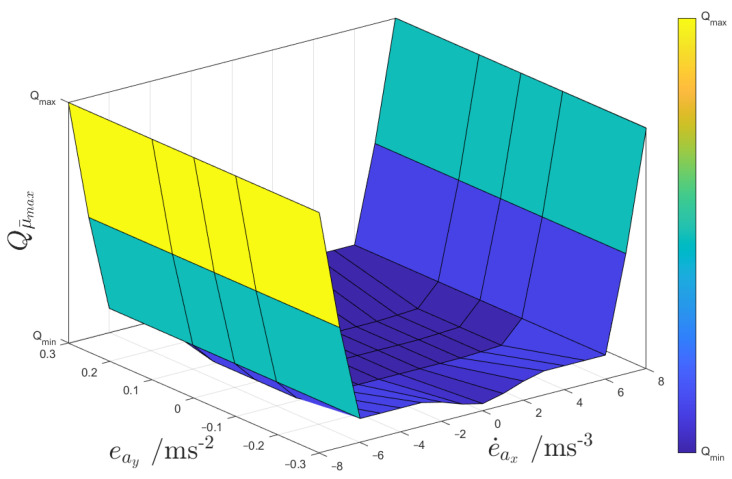
Plot of $Q_{{\bar{\mu }}_{max}}$ as a function of $e_{a_{y}}$ and ${\dot{e}}_{a_{x}}$.

The $Q_{{\bar{\mu }}_{max}}$ look-up table was designed to enhance the responsiveness of the state ${\bar{\mu }}_{max}$ to the actual tyre–road friction level, and to induce rapid reactions of the random walk model. In the generation process, initial one-dimensional maps were generated through brute-force testing, and then were further refined through a gradient-descent optimisation algorithm for each of the purely longitudinal and lateral test scenarios in Section 4.1. The objective was to minimise a cost function based on the relevant key performance indicators (KPIs) in Section 4.2, for the prevalent dynamics excited in the specific manoeuvre. The attained optimal $Q_{{\bar{\mu }}_{max}}$ values were subsequently combined via interpolation to construct the two-dimensional look-up tables. These were further optimised along manoeuvres with combined high slip conditions. The tuning process was repeated for alternative look-up table input variables. $e_{a_{y}}$ and ${\dot{e}}_{a_{x}}$ emerged as the optimal choices. Figure 6 shows the resulting map implemented in UKF ACM.

## 4. Test Scenarios and Key Performance Indicators

### 4.1. Manoeuvres for Performance Assessment

The resulting UKFs were tested: (i) experimentally, in high-friction conditions, at the Lommel proving ground; and (ii) through the high-fidelity CarMaker model, in manoeuvres with very low tyre–road friction levels, which could not be performed within the available experimental facility. Both (i) and (ii) include very challenging conditions from the vehicle dynamics viewpoint. The test scenarios were the following:

- Test scenario 1: experimental 60 m radius skid pad test, in which the vehicle was slowly accelerated while the driver applied steering angle corrections for tracking the reference trajectory, until the car reached the cornering limit, and could no longer stay within the reference lane.
- Test scenario 2: experimental handling circuit lap with the vehicle pushed to its limit by a professional test driver. This test was designed to stress the vehicle at high lateral and longitudinal accelerations, and targeted the UKF performance assessment in peak acceleration conditions.
- Test scenario 3: simulated acceleration and braking test on a very low-friction surface (μ≈ 0.3). The manoeuvre involved the vehicle accelerating from a standstill to a speed of ~ 70 kmh^−1^, followed by heavy braking whereby the anti-lock braking system (ABS) module was activated throughout. A conventional ABS algorithm was chosen, which uses a control law based on the combination of longitudinal tyre slip and wheel deceleration [54]. The ABS was fed with the true values of the relevant variables from the high-fidelity vehicle simulation model, and the two filters received the same inputs from the model, i.e., the simulation results were not affected by the presence of the filters. On the contrary, the traction controller was purposely kept inactive during the acceleration phase, which thus implied significant wheel spinning. The overall test targeted the assessment of the vehicle speed estimation performance in extremely critical conditions.
- Test scenario 4: simulated acceleration test with μ-jump, i.e., with a sudden transition from μ≈ 0.3 to μ≈ 1, which was followed by an immediate acceleration at the vehicle’s maximum capability once the rear wheels had crossed over to the higher friction surface. With the electric motors installed on this vehicle, this equated to a rise in $a_{x}$ from 0 to 6 ms^−2^ in under 0.3 s.
- Test scenario 5: simulated slow sinusoidal steering (with a 0.25 Hz frequency and 45 deg amplitude) manoeuvre at μ≈ 0.3 from an initial speed of 70 kmh^−1^, with the torque demand set to the constant value that would maintain the entry speed if the vehicle was operated in straight line. The steering wheel angle amplitude, $\delta_{swa,max}$, was set to 45 deg, corresponding to a steady-state $a_{y}$ of ~6 ms^−2^. The test focused on the sideslip angle estimation performance in very low-friction conditions.

Although test scenario 3 involved very low-friction conditions, the filters were initialised with ${\bar{\mu }}_{max,0}=$ 1, corresponding to dry tarmac operation; similarly, to make the manoeuvre more challenging following the tyre-road friction transition, in test scenario 4 the initialisation was set to ${\bar{\mu }}_{max,0}=$ 0.3. In all other scenarios, the initialisation was set to ${\bar{\mu }}_{max,0}=$ 1.

Additionally, to ascertain the robustness of the estimators with respect to realistic variations of typical parameters, a sensitivity analysis was conducted on UKF and UKF ACM with ±10% independent changes of the vehicle mass $m_{tot}$, yaw mass moment of inertia $I_{z}$, and tyre cornering stiffness C, for which the changes were made to the equivalent scaling factor of the magic formula model, $\lambda_{ky}$. Due to the inability to change the experimental vehicle demonstrator, the analysis was performed by varying the parameters within the internal model of the filters. For consistency, the same method was applied to the simulation-based analyses.

### 4.2. Key Performance Indicators

The performance of UKF and UKF ACM was analysed through the root mean square (hence, the subscript ‘rms’ in the following notations) values of the error between the UKF estimation outputs and the measured data. Hence, the resulting KPIs are (i) $e_{\beta_{COG},rms}$, the rms value of the estimation error of the sideslip angle at the centre of gravity; (ii) $e_{v_{x},rms}$, the rms value of the longitudinal speed estimation error; (iii) $e_{\sigma_{x,F},rms}$, the rms value of the average estimation error on the slip ratios of the front tyres; and (iv) $e_{\sigma_{x,R},rms}$, the same as (iii), but applied to the slip ratios across the rear axle.

Moreover, for each indicator (i–iv), the relative percentage reduction, ${∆}_{UKF\;ACM|UKF}$, of the estimation error brought by UKF ACM with respect to UKF is computed for nominal internal model parameters. The robustness analyses also include consideration of the following indicators:

(17) $${∆}_{UKF,rp|UKF}=\frac{e_{x,rms,UKF}-e_{x,rms,UKF,rp}}{e_{x,rms,UKF}}100\;{∆}_{UKF\;ACM,rp|UKF}=\frac{e_{x,rms,UKF}-e_{x,rms,UKF\;ACM,rp}}{e_{x,rms,UKF}}100$$

where $e_{x,rms,UKF}$ indicates the KPI value for UKF, related to the estimation of the variable x (with x= $\beta_{COG}$, $v_{x}$, $\sigma_{x,F}$, $\sigma_{x,R}$), for nominal parametrisation of the internal model of the filter; $e_{x,rms,UKF,rp}$ is the KPI value for UKF, referred to the estimation of the variable x, for a variation of the internal model parameter indicated by the subscript ‘rp’ (i.e., robustness parameter, defined such that rp= $m_{tot}$, $I_{z}$, C) in the robustness analysis; and $e_{x,rms,UKFACM,rp}$ is the equivalent of $e_{x,rms,UKF,rp}$ for UKF ACM. Hence, ${∆}_{UKF,rp|UKF}$ and ${∆}_{UKFACM,rp|UKF}$ are the percentage variations of the estimation performance of UKF and UKF ACM with respect to the benchmarking UKF with nominal parametrisation.

## 5. Results

### 5.1. Assessment for Nominal Vehicle Parameters

**Figure 7:**
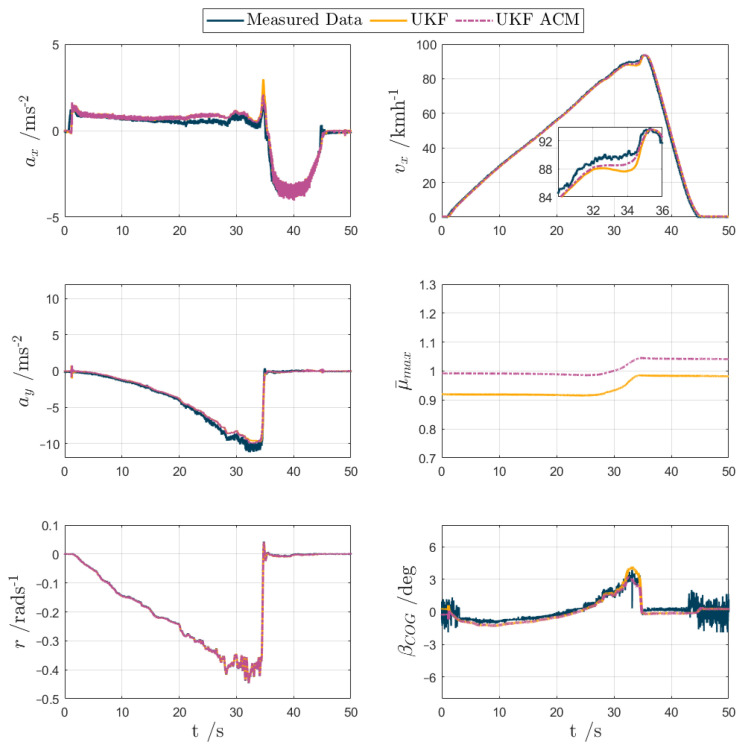
UKF comparison plots for an experimental skid pad manoeuvre (test scenario 1).

**Figure 8:**
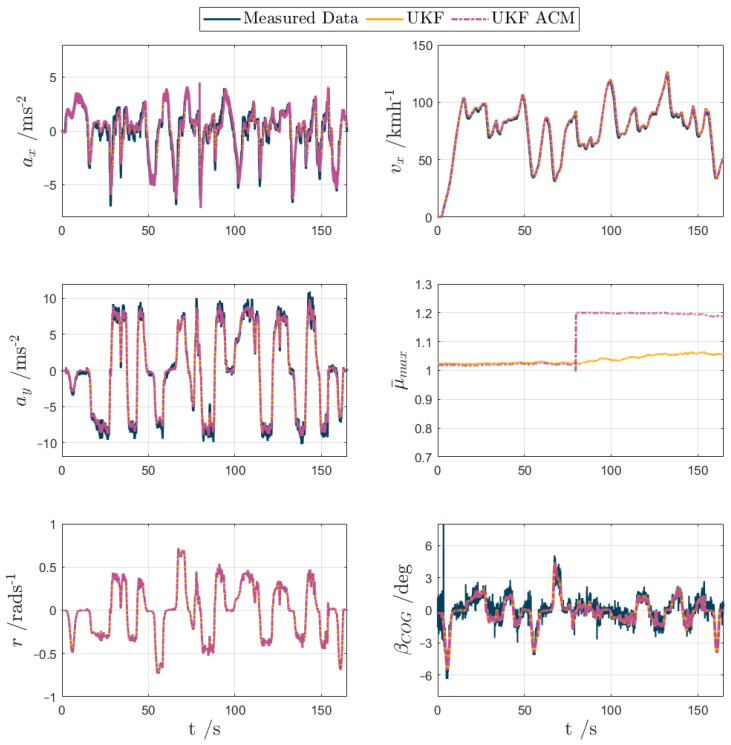
UKF comparison plots for an experimental high-friction handling circuit lap (test scenario 2).

**Figure 9:**
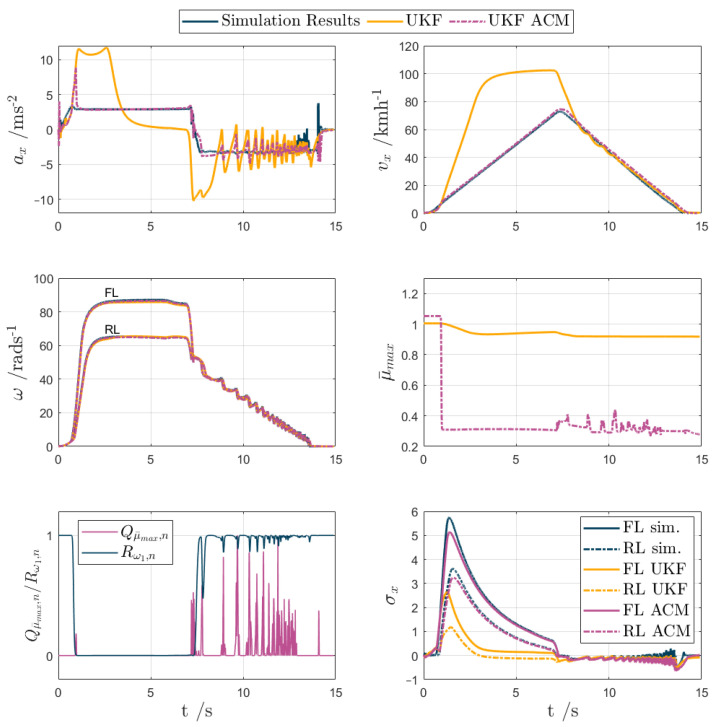
UKF comparison plots for an acceleration and braking test with μ≈ 0.3 (test scenario 3). For the immediacy of notation, the front left and rear left vehicle corners are indicated with ‘FL’ and ‘RL’.

**Figure 10:**
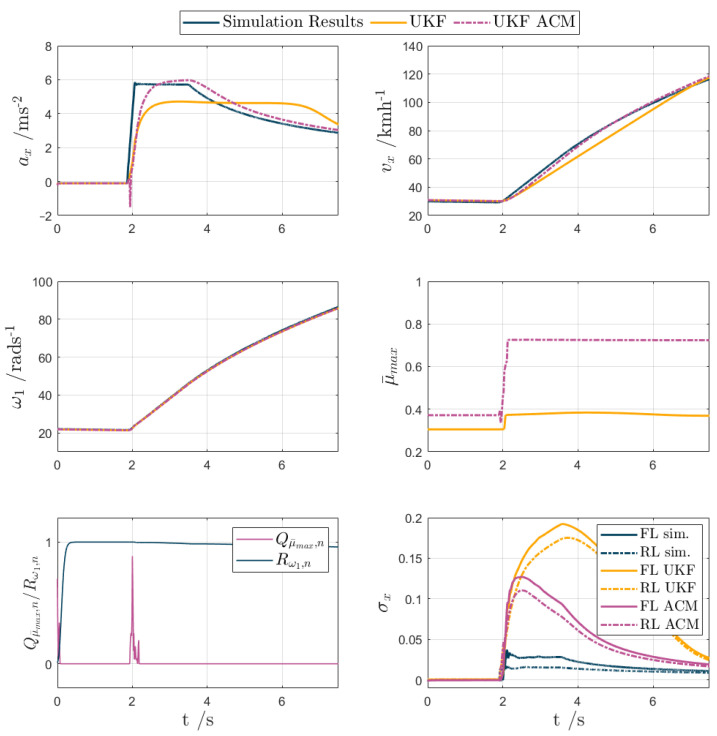
UKF comparison plots for an acceleration test with μ-jump from low to high tyre–road friction conditions (test scenario 4).

**Figure 11:**
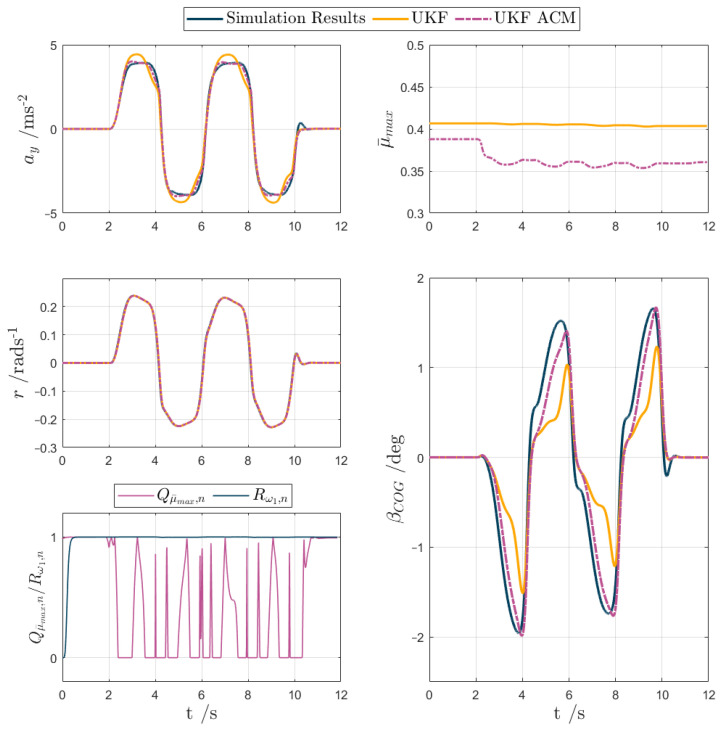
UKF comparison plots for the sinusoidal steering test with μ≈ 0.3 (test scenario 5).

For test scenarios 1–5, the UKF and UKF ACM outputs are compared against the measured data or simulation results in the following Figure 7, Figure 8, Figure 9, Figure 10 and Figure 11, while Table 2 reports the KPIs for all tests. For simplicity of notation, the symbol ‘$\hat{⬚}$’ is omitted from the estimated variables.

Test scenario 1. The maximum measured lateral acceleration achieved in this test exceeded 10 ms^−2^, which implies a real tyre–road friction coefficient slightly above 1. Figure 7 reports the time histories of the main estimated variables and the respective measured profiles. The main useful filter outputs are $\beta_{COG}$ and $v_{x}$. Nevertheless, to assess the overall matching of the estimated vehicle dynamics with the measured behaviour, the figure—similarly to the following ones—also reports additional variables, in this case $a_{x}$, $a_{y}$, and r, which are commonly measured in production vehicles, and ${\bar{\mu }}_{max}$, the auxiliary state that facilitates estimation convergence. Both UKFs estimate the states well in these quasi-steady-state conditions, with some minor differences. For example, UKF ACM outputs a ${\bar{\mu }}_{max}$ profile that is higher—by almost 0.1—than the one from UKF. The increased ${\bar{\mu }}_{max}$ provides modest improvements, of up to 4%, in the estimation of $v_{x}$ and $\beta_{COG}$. This is noticeable at ~34 s, where both UKF ACM estimations are clearly closer to the measured data, e.g., see the inset in the subplot of $v_{x}$. In general, although not being cause for concern in this specific test, the discrepancies between the measurements and the UKF estimates were caused by the model assumptions (e.g., absence of the pitch and heave motions, and the vertical dynamics of the unsprung masses) and mismatches, which tend to be particularly evident in correspondence with the model nonlinearities associated with tyre, suspension, and aerodynamic behaviour.

Test scenario 2. The handling circuit excited the vehicle cornering response in quasi-steady-state and transient conditions, including significant interactions between the longitudinal and lateral dynamics. In fact, $\left|a_{x}\right|$ often exceeded 6 ms^−2^, and $\left|a_{y}\right|$ reached the cornering limit with values of over 10 ms^−2^. In this test, the estimation of $\beta_{COG}$ was paramount for active safety purposes, as it would be used by vehicle stability controllers and other chassis controllers. The same time history plots as in scenario 1 are shown in Figure 8. Aside from a jump in ${\bar{\mu }}_{max}$ at t≈ 80 s, which is attributed to a longitudinal acceleration spike associated with an instance of hard braking, the outputs from the UKF variants are substantially indistinguishable. Both UKFs accurately estimate the sideslip response, with $e_{\beta ,rms}$ values of ~0.5 deg. This level of accuracy was particularly impressive, considering the signal noise magnitude typical of the measurements from the production sensors of the specific vehicle demonstrator, feeding their inputs to the UKFs. Similarly to the first test scenario, UKF ACM brought a minor improvement in all KPIs, with a ~2% reduction of $e_{\beta ,rms}$.

Test scenario 3. This was the first low-friction surface test, in which the ${\bar{\mu }}_{max}$ initialisation at 1 represented a worst-case scenario, given that the actual friction factor was ~0.3. The vehicle accelerated in the first 7.5 s, achieving an $a_{x}$ peak of ~3 ms^−2^, which implied significant wheel spinning given the absence of traction control, before braking to a standstill in approximately the same time, with ABS interventions throughout to prevent wheel locking. Figure 9 reports the time histories of the most relevant variables. On the $a_{x}$ profile, at the beginning of the test, there is a very sharp rise in the $a_{x}$ estimated by the filters, as the driver starts to accelerate and the algorithms have been initialised for high-friction conditions, i.e., they expect higher $a_{x}$ values than those actually measured, which are limited by the surface condition. The error between the accelerations and velocities is high, so $\epsilon_{\omega_{i}}$ drops to its bottom boundary, and subsequently $R_{\omega_{i}}$ (see $R_{\omega_{1},n}$ in the figure, i.e., the normalised $R_{\omega_{1}}$ profile for comparison purposes) increases, to indicate that UKF ACM must give low weight to the measured wheel speeds. This initial transient corresponds to large $\left|{\dot{e}}_{a_{x}}\right|$, which, in turn, at t≈ 1 s, causes a small increase in $Q_{{\bar{\mu }}_{max},n}$, i.e., the normalised value of $Q_{{\bar{\mu }}_{max}}$, responsible for the steep drop in ${\bar{\mu }}_{max}$. After the drop, as the estimated ${\bar{\mu }}_{max}$ matches the real friction level, the wheel speed measurements become relevant again. Unlike UKF, UKF ACM is then able to track the measured $a_{x}$ and $v_{x}$ for the remainder of the acceleration phase with great accuracy. For example, the maximum magnitude of the slip ratio estimation error amounts to ~0.6 for UKF ACM, against ~3.2 for UKF. Both UKF variants estimate $v_{x}$ well during the braking period, although UKF ACM is smoother, whereas UKF staggers during the ABS cycling phase. Correspondingly, the amplitude of the $a_{x}$ oscillations during the braking period is roughly halved by UKF ACM. Overall, across the test, UKF ACM reduces $e_{v_{x},rms}$ by 94%, from 32.3 ms^−1^ to 1.9 ms^−1^, and both $e_{\sigma_{x,F},rms}$ and $e_{\sigma_{x,R},rms}$ by 87%.

The important conclusion is that UKF ACM provided decisively superior $v_{x}$ estimation performance in conditions of very high longitudinal slip. In this and the following tests, the magnitude of the covariance variations for $Q_{{\bar{\mu }}_{max}}$ and $R_{\omega_{i}}$ was significantly higher than for $Q_{r}$, $Q_{v_{x}}$ and $Q_{v_{y}}$, so the latter are not shown in the figures, although they are contributing to the performance improvement. In the experiments for test scenarios 1 and 2, it was also verified that the adoption—within UKF—of a $Q_{{\bar{\mu }}_{max}}$ value facilitating swift variations of ${\bar{\mu }}_{max}$, i.e., corresponding to the top level of $Q_{{\bar{\mu }}_{max}}$ visible in Figure 9 for test scenario 3, would provoke undesirable oscillations of the estimated variables, and unstable filter behaviour during high-friction operation, which confirmed the necessity of the proposed adaptation mechanism.

Test scenario 4. The μ-jump test helped ascertain how well the UKF could detect a swift change in surface, reflecting real-world scenarios, such as road contamination, black ice, or simply transitions from wet to dry tarmac, where accurate estimation, although not being safety-critical, is important not to unnecessarily limit vehicle performance. The results are presented in Figure 10. The road surface changes from low to high μ, and then the vehicle begins to accelerate, just before t = 2 s, with maximum torque demand, which results in an almost step-like increase in $a_{x}$ to ~6 ms^−2^. UKF fails to match the amplitude and resulting shape of the $a_{x}$ curve, as its model expects the vehicle to be in low-friction conditions, whereas UKF ACM is much more prompt. In fact, the rise in $\left|{\dot{e}}_{a_{x}}\right|$ leads to a spike in $Q_{{\bar{\mu }}_{max}}$ which allows for ${\bar{\mu }}_{max}$ to jump from ~0.3 to ~0.75, whereas in UKF, ${\bar{\mu }}_{max}$ only reaches a value of ~0.38. Hence, UKF ACM better matches the $v_{x}$ measurement curve, with $e_{v_{x},rms}$ reducing from ~5 ms^−1^ to ~1.4 ms^−1^, and the peak error value dropping from ~10 ms^−1^ to ~2.5 ms^−1^. With regards to $e_{\sigma_{x,F},rms}$ and $e_{\sigma_{x,R},rms}$, UKF ACM reduces these KPIs by ~57%, with the maximum magnitude of the slip ratio estimation error dropping by ~45%, i.e., from 0.175 to 0.095.

Test scenario 5. In the low-friction sinusoidal steering test (Figure 11), ${\bar{\mu }}_{max}$ was subject to an initial reduction to reasonable levels for both UKF and UKF ACM, occurring at the very beginning of the simulation, when the vehicle accelerated in a straight line to reach the required initial condition, before the relevant section of the manoeuvre reported in the figure. Within the core part of the test, the vehicle exhibits high levels of lateral slip for relatively low $\left|a_{y}\right|$. During each steering transient, UKF overestimates the lateral acceleration peak, and then immediately afterwards underestimates $\left|a_{y}\right|$. The $e_{a_{y}}$ oscillations during the transients provoke the $Q_{{\bar{\mu }}_{max},n}$ oscillations in the respective plot. These cause the UKF ACM ${\bar{\mu }}_{max}$ profile to dip as the vehicle slips laterally, which significantly improves the estimation of the peaks and overall shape of $a_{y}$ and $\beta_{COG}$. For example, the maximum magnitude of the $\beta_{COG}$ estimation error is reduced from over 1 deg to less than 0.3 deg, a 70% improvement compared to UKF. In conclusion, this test demonstrated the enhanced performance of UKF ACM in its $\beta_{COG}$ estimation on low-μ surfaces. Minor improvements, amounting to less than 5%, were also seen in $e_{v_{x},rms}$, $e_{\sigma_{x,F},rms}$, and $e_{\sigma_{x,R},rms}$.

### 5.2. Robustness Analysis

**Figure 12:**
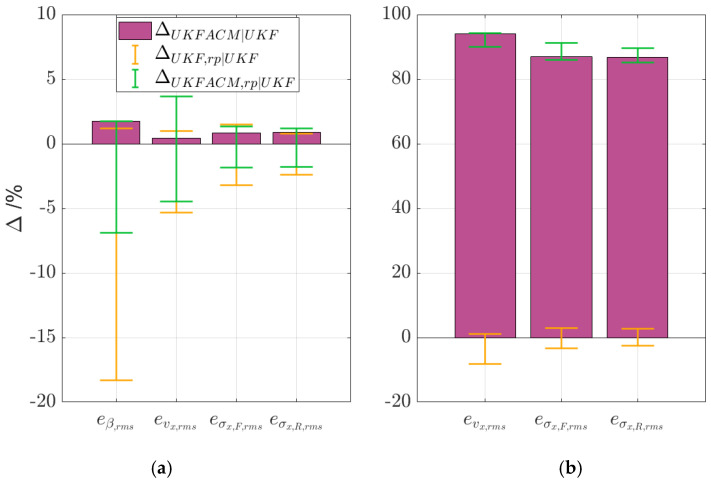
Histograms of ${∆}_{UKF\;ACM|UKF}$, and error bars for ${∆}_{UKF,rp|UKF}$ and ${∆}_{UKF\;ACM,rp|UKF}$, for the considered robustness analyses, along (**a**) the experimental handling circuit (test scenario 2); and (**b**) the simulated acceleration and braking test in low-friction conditions (test scenario 3).

Figure 12 summarises the results of the robustness analysis along test scenarios 2 and 3 (see also Table A1 in the Appendix A for the full dataset). In the plots, the histograms refer to ${∆}_{UKF\;ACM|UKF}$, while the error bars represent the range of variation of ${∆}_{UKF,rp|UKF}$ and ${∆}_{UKF\;ACM,rp|UKF}$, across the sensitivity checks.

The histograms in subplot (a) show that, on the handling circuit in high-friction conditions and with nominal internal model parametrisation, the improvements brought by UKF ACM are marginal (~2% on average) but consistent across the KPIs. The top and bottom caps of the error bars of UKF ACM are all at a higher level than the respective ones for UKF, which corresponds to better performance for the considered parameter range. This is confirmed in Table A1, in which all UKF ACM KPIs are better than those for UKF with the same internal model mismatch. For example, UKF occasionally performs worse by nearly 20% in terms of $e_{\beta ,rms}$ with respect to its nominal case, particularly when altering the tyre cornering stiffness. The maximum $e_{\beta ,rms}$ performance decay of the UKF ACM with internal model mismatch with respect to the nominal UKF is only 7%. With the exception of that of $e_{v_{x},rms}$, UKF ACM has shorter error bars than UKF, which represents a smaller spread of values, and as such is symptomatic of robustness. Notably, the error bar for $e_{v_{x},rms}$ is longer for UKF ACM, but the increased spread is only in the positive range, where the filter with covariance adaptation markedly improves the estimation.

For test scenario 3 (see subplot (b)), the spread of $e_{\sigma_{x,F},rms}$ and $e_{\sigma_{x,R},rms}$ is roughly the same for both UKF variants, while the one for $e_{v_{x},rms}$ is lower for UKF ACM. Nevertheless, the difference in estimation accuracy is so wide that the error bars for the two filter variants do not have any overlap.

## 6. Conclusions

This paper presented a novel vehicle state estimator, i.e., the so-called UKF ACM, of the vehicle longitudinal and lateral components of the vehicle velocity, tyre slip ratios, as well as sideslip and slip angles. The novelty lies in the adaptive formulations of the process and measurement noise covariances. For fairness of assessment, the fixed covariances of a well-calibrated baseline UKF were also used as the baseline values for UKF ACM. The two UKF variants were compared in a variety of experimental and simulated manoeuvres, including very low tyre–road friction conditions as well as longitudinal and lateral dynamics.

Based on the results in Section 5, the main conclusions are the following:

- In high-friction conditions near the limit of handling, the performance of the baseline UKF with fixed covariances was very similar to that of UKF ACM, with the latter providing small but still noteworthy benefits.
- In extreme longitudinal slip cases on low μ with highly incorrect friction level initialisation within the filters, UKF ACM performed significantly better than UKF. In fact, unlike UKF, the variations in the process and measurement noise covariances $Q_{{\bar{\mu }}_{max}}$ and $R_{\omega_{i}}$, related to the friction random walk model and wheel speed measurements, enabled UKF ACM to promptly detect the actual tyre–road friction level, and achieve highly accurate speed and slip ratio estimation. Similarly, UKF ACM was very effective in identifying instantaneous and extreme changes in μ, with the related positive impact in terms of the resulting estimation.
- In extreme lateral slip conditions on very low μ surfaces, the increased sensitivity of ${\bar{\mu }}_{max}$ of UKF ACM allowed it to outperform the baseline UKF, with improvements of over 50% in $e_{\beta ,rms}$. This highlighted a clear safety improvement, as accurate sideslip angle estimation is necessary for typical vehicle chassis controllers.
- UKF ACM has shown notable robustness with respect to UKF. In fact, when varying—within the internal models of the filters—parameters that would realistically change during real-world vehicle operation, (i) the UKF ACM KPI decay was maintained within the tolerable range of 10% of the original values for nominal conditions, while UKF experienced a maximum performance reduction that approached 20%; and (ii) the UKF ACM results were the same or better, e.g., by more than 80% in the high longitudinal tyre slip conditions of test scenario 3, than the corresponding UKF ones.

Future work will include (i) experimental testing on surfaces with low and variable tyre–road friction conditions; (ii) the production-oriented implementation of UKF ACM on automotive control hardware; (iii) the integration of machine learning algorithms in the UKF adaptation scheme; and (iv) the comparison of the proposed formulation with the most promising machine learning techniques for vehicle dynamics state estimation.

## Figures and Tables

**Table 1:** Main parameters of the case study vehicle.

Description	Symbol	Value	Unit
Vehicle mass in real testing conditions	$m_{tot}$	2843	[kg]
Yaw mass moment of inertia	$I_{z}$	4124	[kgm^2^]
Front semi-wheelbase	a	1.47	[m]
Rear semi-wheelbase	b	1.46	[m]
Front track width	$t_{f}$	1.60	[m]
Rear track width	$t_{r}$	1.60	[m]
Centre of gravity height	$h_{COG}$	0.63	[m]
Wheel radius	R	0.38	[m]

**Table 2:** KPIs for UKF and UKF ACM along the considered test scenarios.

	$e_{\beta ,rms}$	${∆}_{UKF\;ACM|UKF}$	$e_{v_{x},rms}$	${∆}_{UKF\;ACM|UKF}$	$e_{\sigma_{x,F},rms}$	${∆}_{UKF\;ACM|UKF}$	$e_{\sigma_{x,R},rms}$	${∆}_{UKF\;ACM|UKF}$
	[deg]	[%]	[ms^−1^]	[%]	[−]	[%]	[−]	[%]
Test Scenario 1—Experimental skid pad								
UKF	0.429	-	1.531	-	0.0264	-	0.0287	-
UKF ACM	0.415	−3.15%	1.472	−3.83%	0.0257	−2.54%	0.0282	−1.84%
Test Scenario 2—Experimental handling circuit								
UKF	0.510	-	2.515	-	0.0157	-	0.0173	-
UKF ACM	0.501	−1.76%	2.504	−0.43%	0.0156	−0.83%	0.0171	−0.87%
Test Scenario 3—Simulated acceleration and braking with μ≈ 0.3								
UKF	-	-	32.254	-	1.3988	-	1.0325	-
UKF ACM	-	-	1.925	−94%	0.1828	−87%	0.1361	−87%
Test scenario 4—Simulated acceleration with μ-jump								
UKF	-	-	4.993	-	0.0956	-	0.0928	-
UKF ACM	-	-	1.373	−72%	0.0422	−56%	0.0401	−57%
Test scenario 5—Simulated sinusoidal steering test with μ≈ 0.3								
UKF	0.508	-	0.985	-	0.0327	-	0.0328	-
UKF ACM	0.233	−54%	0.948	−3.71%	0.0322	−1.41%	0.0323	−1.40%

## Data Availability

The data presented in this study are available on request from the corresponding author.

## Appendix A

**Table A1:** KPIs for the robustness analysis for test scenarios 2 and 3.

	$e_{\beta ,rms}$	$e_{v_{x},rms}$	$e_{\sigma_{x,F},rms}$	$e_{\sigma_{x,R},rms}$
	[deg]	[ms^−1^]	[−]	[−]
Test scenario 2—Experimental handling circuit				
UKF	0.510	2.515	0.0157	0.0173
UKF $m_{tot}$+10%	0.571	2.644	0.0157	0.0175
UKF $m_{tot}$–10%	0.511	2.491	0.0158	0.0171
UKF $I_{z}$+10%	0.521	2.515	0.0157	0.0173
UKF $I_{z}$−10%	0.504	2.514	0.0157	0.0173
UKF C+10%	0.537	2.489	0.0155	0.0171
UKF C–10%	0.604	2.535	0.0162	0.0177
UKF ACM	0.501	2.504	0.0156	0.0171
UKF ACM $m_{tot}$+10%	0.541	2.639	0.0157	0.0174
UKF ACM $m_{tot}$–10%	0.525	2.421	0.0157	0.0170
UKF ACM $I_{z}$+10%	0.524	2.504	0.0157	0.0173
UKF ACM $I_{z}$−10%	0.516	2.504	0.0157	0.0173
UKF ACM C+10%	0.525	2.503	0.0154	0.0170
UKF ACM C–10%	0.554	2.529	0.0162	0.0177
Test scenario 3—Simulated acceleration and braking with μ≈0.3				
UKF	-	32.254	1.3988	1.0325
UKF $m_{tot}$+10%	-	31.660	1.3575	1.0047
UKF $m_{tot}$–10%	-	34.876	1.4454	1.0576
UKF $I_{z}$+10%	-	32.253	1.3988	1.0325
UKF $I_{z}$−10%	-	32.276	1.4006	1.0337
UKF C+10%	-	32.211	1.4015	1.0376
UKF C–10%	-	32.440	1.4161	1.0441
UKF ACM	-	1.925	0.1828	0.1361
UKF ACM $m_{tot}$+10%	-	3.153	0.1489	0.1339
UKF ACM $m_{tot}$–10%	-	3.195	0.1960	0.1527
UKF ACM $I_{z}$+10%	-	2.449	0.1357	0.1085
UKF ACM $I_{z}$−10%	-	1.895	0.1745	0.1296
UKF ACM C+10%	-	1.886	0.1744	0.1305
UKF ACM C–10%	-	1.994	0.1874	0.1395
